# Editorial: Conference Research Topic: 9th symposium on Antimicrobial Resistance in Animals and the Environment (ARAE 2023)

**DOI:** 10.3389/fmicb.2024.1509192

**Published:** 2024-10-22

**Authors:** Benoît Doublet, Sébastien Olivier Leclercq, Michel Stanislas Zygmunt, Axel Cloeckaert

**Affiliations:** ISP, INRAE, Université de Tours, Nouzilly, France

**Keywords:** epidemiology, resistance mechanisms, microbiota, whole genome sequencing, antibiotics

The international symposium on Antimicrobial Resistance in Animals and the Environment (ARAE) is a renowned scientific event gathering many scientists in the field from all over the world every 2 years since 2005. Its 9th edition was held in July 2023 in Tours, France. The aim of the ARAE conference is to present an up-to-date vision of the impact of antibiotic use and resistance in the animal setting, its environment and subsequent impact on human health. All aspects related to epidemiology of antibiotic-resistant strains, genetic spread of antimicrobial resistance genes, emerging resistance mechanisms, resistome in microbiota, and the role of the environment as dissemination route and potential reservoir for resistance genes acquisition are discussed. The 9th edition of ARAE welcomed 170 registered participants from 23 countries (Australia, Belgium, Canada, China, Czech Republic, Denmark, Finland, France, Germany, Ireland, Israel, Italy, Japan, Netherlands, Norway, Philippines, Poland, Portugal, South Korea, Spain, Tunisia, United Kingdom, United States). Early-career scientists represented almost 40% of participants. Five topic sessions gathered 59 oral presentations including keynote lectures and 94 research studies presented as posters. The proceedings of this 9th ARAE symposium can be found at https://hal.inrae.fr/hal-04170355. The present conference Research Topic offered the opportunity to 150 authors worldwide to publish their studies presented during the conference as well as for scientists who were unable to attend the ARAE symposium. It includes one review, one systematic review and 16 original research articles.

First, in an original review, Frederiksen et al. addressed the current knowledge on polyether ionophore resistance and the potential consequences in a One Health perspective. Polyether ionophores are largely used as feed additives in poultry production worldwide, especially monensin, to control avian coccidiosis due to *Eimeria* spp. Beside anti-parasitic activity, polyether ionophores has antibacterial activity but are not used in human medicine due to their toxicity. While Gram-negative bacteria are generally intrinsically resistant to polyether ionophores, Gram-positive bacteria such as *Enterococcus faecium* have been reported to harbor plasmid-borne *narAB* resistance genes against narasin with a yet unknown mechanism. Currently, there are sparse evidences of cross-resistance between ionophoric antibiotics and critically-important antibiotics for human medicine. However, the *narAB* resistance genes have been found to co-localize on conjugative plasmids of *E. faecium* with antibiotic resistance genes to macrolides, tetracycline and glycopeptides. This suggests that the use of narasin in broiler production systems could co-select vancomycin-resistant *Enterococci* that can pose a threat to human health.

*Salmonella enterica* spp. are important zoonotic pathogens related to foodborne diseases worldwide. Multidrug-resistant (MDR) *Salmonella* spp. have been classified by the World Health Organization as high priority pathogens for which new antibiotics are urgently needed. Here, Yan et al. carried out a bibliometric analysis to document trends in past and current researches dealing with horizontal gene transfer implicated in antimicrobial resistance spread in bacterial isolates of *S. enterica*. Since 1999, the number of publications in this field has shown an increasing trend with more than 100 publications per year in the recent years. There has been an evolution of research hotspots from (i) understanding of multidrug resistance in *S. enterica* serovar Typhimurium DT104 and other *S. enterica* serovars, (ii) emergence of plasmid-mediated expanded-spectrum cephalosporin resistance, to (iii) the analysis of whole genome sequences that has significantly enriched our understanding of the population structure, transmission dynamics, and epidemiology of antimicrobial resistance in *S. enterica*. Recent methods such as high-throughput long-read sequencing will provide exciting opportunities to deepen scientific understanding in this research domain.

This conference Research Topic included also three articles focused on recent methodologies for rapid typing of MDR bacterial isolates and to harmonize methodologies in veterinary clinical laboratories. Zendri, Schmidt et al. assessed the potential of the InfraRed spectroscopy using the IR Biotyper for typing of nosocomial outbreaks of MDR pathogens in veterinary hospitals. They compared InfraRed spectroscopy and whole genome analysis of retrospective collections of *Klebsiella pneumoniae* and *Pseudomonas aeruginosa* isolated from companion animals (dogs and cats) and horses in two veterinary hospitals. InfraRed spectroscopy revealed a significant discriminatory power for *K. pneumoniae* isolates to identify clonal transmission events within veterinary hospital settings. However, this methodology appears less accurate for typing veterinary *P. aeruginosa* isolates indicating that further optimization is needed before applying in routine veterinary laboratories. In another research article, dealing with bacterial morphology and antibiotic resistance, Ikebe et al. reported morphological differences using light microscopy between laboratory-evolved antibiotic-resistant *E. coli* strains and their susceptible parental ones. They correlated morphological features of resistant strains with phenotypic resistance and gene expression changes in energy metabolism and multidrug efflux systems. They proposed a novel image-based deep learning method for single-cell classification between resistant and susceptible strains. Further improvements of such deep learning algorithm would probably enable the identification of resistant bacterial cells using light microscopy in a near future. Finally, Koritnik et al. reported the results of a European survey about methodologies in veterinary microbiology laboratories (*n* = 241 labs from 34 European countries). They highlighted a broad diversity of methods for bacterial culture and identification with result reports ranging from 2 to 8 days. In European veterinary microbiological diagnostic laboratories, disc-diffusion method and minimal inhibitory concentration determination were both used for antimicrobial susceptibility testing as well as EUCAST and CLSI clinical breakpoints as interpretative criteria. This large European survey clearly emphasizes that harmonization is needed in bacterial culture and identification and in antimicrobial susceptibility testing for reporting and comparison purposes.

A large set of original research articles (*n* = 7) concerned molecular epidemiology of antimicrobial-resistant bacteria, mainly *E. coli*, isolated from food-producing animals, food, horses and veterinary hospitals. Antimicrobial resistance (AMR) is an important One Health issue in broiler production worldwide, notably by the carriage of MDR bacteria in the gut of healthy animals that can contaminate meat (Seiffert et al., 2013). Leclercq et al. performed an in depth genomic analysis of MDR *E. coli* isolated in an experimental facility reproducing the entire broiler production pyramid without antibiotic use since more than 10 years. They demonstrated that no transmission of MDR *E. coli* occurred from hens to offspring nor acquisition at the hatchery during three generations, but that the downstream rearing environment may constitute the source of few MDR *E. coli* clonal populations able to colonize young chicks. In addition, they showed a strong association between each major *E. coli* clonal populations and their own MDR IncF plasmid subtypes over chicken generations. Davies et al. reported a study focused on commensal *E. coli* broilers from live bird markets in Bangladesh. They described an extremely high occurrence of genetically-diverse MDR *E. coli* from caecal samples (93%). While ciprofloxacin resistance was very common, other resistances to critically-important antibiotics remained rare or absent (cephalosporins, carbapenems, colistin) with very few plasmid-borne resistance genes [*bla*_CTX−M_, *mcr*-*1, fosA, tet*(X)]. In another study, Dixit et al. assessed the diversity of resistant bacteria in raw chicken and pork meat samples in Australia. They identified 33 bacterial species belonging to 17 genera. Among a total of 288 isolates, 12% were phenotypically MDR. Chicken meat samples carried more MDR isolates than pork samples. WGS analysis of all isolates revealed a large diversity of AMR genes, few ones being considered of critically importance for human medicine.

Resistance to expanded spectrum cephalosporins in veterinary medicine is a major concern for human health (Seiffert et al., 2013). Leoni et al. investigated the occurrence of Extended-Spectrum β-Lactamase (ESBL)-producing *E. coli* recovered from clams of the Central Adriatic coast in Italy, from 2018 to 2019. They reported a prevalence of 3% genetically-diverse ESBL-producing *E. coli*, the *bla*_CTX−M_ resistance genes being the most prevalent. The 13 ESBL/AmpC-producing *Escherichia* spp. recovered from seven out 28 sampling points were of various sequence types and phylogroups suggesting that various microbiological pollution sources can contaminate the surrounding waters of bivalve areas. Zendri, Isgren et al. reported a surveillance pilot study of Gram-negative isolates resistant to expanded spectrum cephalosporins in two veterinary hospitals. During a 6 months sampling campaign, they highlighted the important occurrence of ESKAPE Gram-negative bacteria in companion animals (equine and small animals) in carriage, clinical and environmental samples. Specific AMR genotypes of *Enterobacter cloacae* complex carrying SHV and or TEM variants and *P. aeruginosa* carrying OXA-50 were prevalent among the equine hospital (horses and environment), while *K. pneumoniae* isolates harboring SHV variants and DHA-1 were only found in the small animal hospital. Although, it remains difficult to conclude on transmission routes, their results strongly suggested that intra-hospital transmission occurred for certain ESC-resistant ESKAPE pathogens. Similarly, in a large epidemiological retrospective study dealing with *K. pneumoniae* in France, Gravey et al. performed a genomic investigation of MDR and hypervirulence of equine isolates between 1996 and 2020. They described a high diversity of *K. pneumoniae* genotypes by MLST and cgMLST including few isolates characterized as MDR and hypervirulent, thus considered as high-risk clones. These STs originate from France but had never been described as MDR-hypervirulent. Plasmid plasticity and horizontal transfer among *K. pneumoniae* populations constitute the risk of emergence of MDR or MDR-hypervirulent high risk clones. Finally, de Lagarde et al. evaluated the impact of a new legislation implemented in 2019 in Québec (Canada) to limit the use of medically-important antibiotics in food-producing animals. They assessed the carriage of ESBL/AmpC-producing *E. coli* in dairy farms before and after the implementation of this new legislation. They described some clonal lineages carrying various AMR genes (*bla*_CTX−M_, *bla*_SHV_, *qnr*…) that persisted over the 4-year period and are disseminated across different dairy farms.

This conference Research Topic includes also three research articles based on metagenomic approach as tools to analyse microbiota diversity and resistome in various settings. Herman et al. evaluated long-read metagenomic sequencing for the detection of bovine respiratory pathogens (*Mannheimia haemolytica, Pasteurella multocida*, and *Histophilus somni*) from deep nasopharyngeal swab samples of calves. Non-selective bacterial enrichment increased 2–4 orders of magnitude the detection of pathogens as well as their AMR genes to macrolides, sulfonamides and tetracyclines; three antibiotics used for bovine respiratory disease management. Third generation metagenomic sequencing could represent a valuable diagnostic tool for pathogen detection and antimicrobial stewardship. In another study dealing with beef cattle production, Strickland et al. investigated the impact of feed supplementation with tylosin and/or probiotics on the microbial communities within feces, manure and airborne particulate matter. They highlighted different abundances of bacterial populations according to the type of samples and the sampling time, but no change in bacterial community compositions of feces, manure and particulate matter related to treatments, i.e., antibiotics and/or probiotics. Naudin et al. developed an elegant *in vitro* model of biofilm to study the impact of fluoroquinolones on the microbiota composition of sewer biofilms. They demonstrated that biofilm exposure to a low concentration of fluoroquinolones mimicking fluroquinolone concentrations typically found in wastewater had no effect on biofilm diversity nor on the relative abundance of fluoroquinolone mutations in QRDR. While high exposure to fluoroquinolones decreased diversity in *in vitro* biofilms and increased the abundance of ciprofloxacin-resistant bacteria including *E. coli* harboring multiple QRDR mutations. Their results suggested that sewer biofilms may constitute a reservoir of fluoroquinolone-resistant bacteria that can subsequently disseminate in the downstream environment.

Resistance acquisition is based on the horizontal transfer of mobile resistance genes or on mutation acquisitions in chromosomal genes encoding antibiotic targets or regulatory factors (Munita and Arias, 2016). Two research articles investigated the role of the *ramRA* multidrug efflux regulatory locus in acquisition of doxycycline resistance in *K. pneumoniae* and in repression of invasiveness of *S. enterica* serovar Typhimurium. Kenyon et al. described the acquisition of frameshift mutations in the *ramR* gene of *K. pneumoniae* during *in vitro* doxycycline selection and in an *in vivo Galleria mellonella* infection model treated with doxycycline. Frameshift inactivation of *ramR* resulted in 24–48 and over 10-fold increases in doxycycline and ciprofloxacin MICs, respectively. Besides, Giraud et al. investigated the role of *ramRA* regulatory locus in response to bile salts on the *Salmonella* cellular invasiveness. They demonstrated that bile-mediated repression of *S*. Typhimurium invasion is partly regulated by major primary bile salts known to activate *ramA* transcription by interacting with RamR, but also that other unknown pathways likely play a role in bile-mediated repression of invasion independently of the *ramRA* locus.

Finally, Imazaki et al. investigated the minimal selective concentration of oxytetracycline using two isogenic resistant/susceptible strains of *E. coli* in Mueller-Hinton medium and in sterilized intestinal contents of White large pigs. This original comparison showed that the sub-MIC selective window (range between minimal selective concentration and MIC) was lower in Muller-Hinton broth compared to sterilized intestinal contents suggesting that intestinal content composition reduce free oxytetracycline proportion. These findings may have implications in understanding of emergence of AMR in gut as well as for antibiotic administration dosage.

To conclude, this conference Research Topic partly illustrated the diversity of research area related to antimicrobial resistance in the field of animals and the environment. The next edition of ARAE (https://arae2025.de) will be held in Berlin, Germany in July 2025, and chaired by Prof. Stefan Schwarz (Institute of Microbiology & Epizootics of the Free University of Berlin).
